# A qualitative study on newly settled migrants’ perceptions of civic and health orientation in Sweden

**DOI:** 10.1093/eurpub/ckac130.141

**Published:** 2022-10-25

**Authors:** M Al-Adhami, K Hjelm, J Wångdahl, E Larsson

**Affiliations:** 1 SWEDESD, Department of Women's and Children's Health, Uppsala, Sweden; 2 CHAP, Department of Public Health & Nursing Science, Uppsala, Sweden; 3 Caring Sciences, Department of Public Health & Nursing Science, Uppsala, Sweden; 4 GloSH, Department of Global Public Health, Stockholm, Sweden

## Abstract

**Background:**

Migrants face structural, socio-political barriers in their resettlement processes that affect their health. Migration also impacts resources such as social capital and health literacy that are of importance for health and integration in society. Hence, there is a need for health promotion in the post-migration phase. The aim of the study was to explore participants' perceptions and experiences of civic orientation with added health communication.

**Methods:**

Six focus group discussions were performed with 32 men and women. Two were held in Arabic, two in Farsi and two in Somali, with native speaking moderators. Participants were recruited from civic orientation classes in the county of Stockholm. An interview guide with semi-structured questions was used. The data were analysed using a method for content analysis of focus group discussions.

**Results:**

Three main categories were identified: ‘The course gives valuable information but its delivery needs adjustments', which includes that the civic and health orientation is valuable and needed earlier, during asylum phase, and that planning and course content need adjustments. ‘The health communication inspired participants to focus on their health', includes that the health communication was useful and inspired uptake of healthier habits. ‘Participation in the course promoted independence and self-confidence', which includes that the course gave insights into society and values in Sweden and promoted independence and new social contacts.

**Conclusions:**

This study adds knowledge of the users' perspectives on the potential of civic orientation to promote health and integration of newly settled migrants, describing ways in which civic orientation with added health communication prompted health and empowerment. However, the content and delivery of the course needs adjustment to better fit the migrants' life situations and varying pre-existing knowledge.

**Key messages:**

- The civic and health information is needed early in the resettlement phase.
- Participation in the course promoted health awareness, independence and self-confidence.

